# Magnetic gelatin-hesperidin microrobots promote proliferation and migration of dermal fibroblasts

**DOI:** 10.3389/fchem.2024.1478338

**Published:** 2024-10-10

**Authors:** Xuyan Sun, Hua Yang, Han Zhang, Weiwei Zhang, Chunyu Liu, Xiaoxiao Wang, Wenping Song, Lin Wang, Qingsong Zhao

**Affiliations:** ^1^ The Fourth Affiliated Hospital of Harbin Medical University, Harbin, China; ^2^ Department of Obstetrics and Gynecology, National Clinical Research Center for Obstetric & Gynecologic Diseases, State Key Laboratory of Common Mechanism Research for Major Diseases, Peking Union Medical College Hospital, Chinese Academy of Medical Sciences and Peking Union Medical College, Beijing, China; ^3^ School of Biomedical Sciences, Harbin Medical University, Harbin, China; ^4^ School of Mechanical and Power Engineering, Zhengzhou University, Zhengzhou, China; ^5^ Wanjia Compulsory Isolation and Drug Rehabilitation Hospital, Harbin, China; ^6^ State Key Lab of Robotic and System, Harbin Institute of Technology, Harbin, China

**Keywords:** microrobots, gelatin-hesperidin, fibroblasts, diabetic foot ulcer, wound healing

## Abstract

Dermal fibroblasts play a crucial role in the formation of granulation tissue in skin wounds. Consequently, the differentiation, migration, and proliferation of dermal fibroblasts are considered key factors in the skin wound healing process. However, in patients with diabetic foot ulcers, the proliferation and migration of fibroblasts are impaired by reactive oxygen species and inflammatory factors impair. Therefore, a novel magnetic gelatin-hesperidin microrobots drug delivery system was developed using microfluidics. The morphology, motility characteristics, and drug release of the microrobot were assessed, along with its impact on the proliferation and migration of human dermal fibroblasts under high-glucose conditions. Subjected to a rotating magnetic field, the microrobots exhibit precise, controllable, and flexible autonomous motion, achieving a maximum speed of 9.237 μm/s. *In vitro* drug release experiments revealed that approximately 78% of the drug was released within 30 min. It was demonstrated through cellular experiments that the proliferation of human dermal fibroblasts was actively promoted by the nanorobot, the migration ability of fibroblasts in a high-glucose state was enhanced, and good biocompatibility was exhibited. Hence, our study may provide a novel drug delivery system with significant potential for promoting the healing of diabetic foot wounds.

## 1 Introduction

Diabetic foot ulcer (DFU) is among the most severe complications of diabetes mellitus (DM) (Saeedi et al., 2020), presenting a significant risk of disability and mortality (Everett and Mathioudakis, 2018). The essence of DFU is a foot ulcer that arises due to diminished blood supply to the foot, as well as numbness and altered sensation resulting from a combination of vasculopathy and nerve damage in the lower extremities under conditions of persistent hyperglycemia (Huang et al., 2023). Currently, the standard clinical treatment for chronic diabetic foot wounds includes thorough debridement, infection control, blood glucose control, and wet dressing changes. However, some studies have indicated that the cure rate of chronic diabetic foot wounds at 12–20 weeks remains less than 50% even with a comprehensive treatment approach (Apelqvist et al., 1993). Therefore, finding effective treatment methods for curing DFUs is particularly important (Wong et al., 2015). Fibroblasts play a crucial role in the formation of granulation tissue (Huang et al., 2020). However, it has been demonstrated that the body reduces the migratory force of fibroblasts under hyperglycemia by affecting the NF-κB-JNKs pathway (Xuan et al., 2016), and that fibroblasts isolated from the ulcers of patients have abnormal morphology, slow proliferation, and exhibit abnormal protein secretion compared to normal fibroblasts in the control group (Pang et al., 2016). Therefore, promoting the cell survival and migration capacity of fibroblasts is a key factor for successful wound healing in DFU(Voza et al., 2024). Hesperidin is a flavonoid commonly present in citrus fruits (AL-Ishaq et al., 2019), It has been previously reported to possess not only hypoglycemic and insulin-sensitizing effects, but also a range of beneficial properties including lipid-lowering, antioxidant, anti-cancer, anti-inflammatory activities. Wang et al. observed that hesperidin decreased the expression of inflammatory mediators at the wound site (Dokumacioglu et al., 2019), augmented the expression of growth-related factors, and boosted angiogenic activity, ultimately facilitating the healing process of diabetic foot ulcers (Wang et al., 2018). However, due to its poor water solubility and low oral bioavailability (Dokumacioglu et al., 2019), hesperidin is not extensively utilized in clinical practice (Li and Schluesener, 2017). Thus, the development of an innovative drug delivery system to enhance the utilization of hesperidin represents a promising approach for treating diabetic foot. Accordingly, we aim to load hesperidin onto a microrobotic drug delivery system to achieve efficient utilization and controlled release of the drug (Marui et al., 2007).

Currently, research on micro-nanodrug carriers is currently gaining traction (Qin et al., 2022). This technology employs precise micro-nanoscale dimensions and structures to deliver active pharmaceutical ingredients (APIs) with greater precision to their intended sites of action (Shen et al., 2023). Such drug delivery systems enhance drug circulation within the body (Xu et al., 2023), improve the pharmacokinetic profile of the drug, and mitigate the occurrence of adverse drug reactions (Bilia et al., 2014). The microrobots are propelled by a magnetic driving field, enabling targeted drug delivery to specific body regions (Shvedova et al., 2003). Precise control over the speed and direction of these microrobots is achieved by controlling the direction and magnitude of the applied magnetic field (Li et al., 2023). Furthermore, low-intensity magnetic fields are both harmless and environmentally friendly (Sitti and Wiersma, 2020), and they are currently extensively employed in nanomedicine applications (Echave et al., 2017). The magnetic drive method is among the most commonly utilized approaches for propelling microrobots (Xu et al., 2021). Magnetic-driven microrobots are distinguished by their high speed, precise controllability, extensive degrees of freedom, and wireless, fuel-free propulsion (Yu et al., 2019). This experiment provides a detailed description of the fabrication process for magnetic gelatin-hesperidin microrobots, as well as their directed movement propelled by a three-dimensional rotating magnetic field generator. This microrobot can accurately navigate around the lesion and release the encapsulated drug within the blood vessel under the influence of a peripheral magnetic field.

In summary, this study successfully designed a novel magnetically driven gelatin-hesperidin microrobot drug delivery system utilizing microfluidic technology. In the experiment, a self-constructed electromagnetic coil system was employed to accurately control the motion of the drug-carrying microrobot, enabling targeted and precise drug release. Gelatin serves as an encapsulating agent for the drug, facilitating a slowly release effect to enhance the pharmacokinetic profile of hesperidin. Additionally, we analyzed the geometry, trajectory, and performance of the drug-carrying robot. Furthermore, we compared the effects of hesperidin-gelatin microrobots with those of hesperidin administration alone on the proliferation and migration potential of fibroblasts under high glucose conditions. In conclusion, this experiment elucidates a novel and promising fibroblast-based therapeutic approach for facilitating diabetic foot wound healing.

## 2 Materials and methods

### 2.1 Synthesis of magnetic gelatin-hesperidin microrobots

#### 2.1.1 Fabrication of microfluidic chips

The microfluidic chip device is comprised of Polydimethylsiloxane (PDMS), capillary stainless steel tubes, dispensing needles, and plastic capillary hoses. The plastic capillary hoses are used as microchannels for dispersed-phase and continuous-phase. One end of the capillary stainless steel tube is linked to the aperture of the PDMS unit, while the other end is attached to the plastic capillary hose for accessing the dispersed and continuous phase fluids. The exit of the PDMS unit is coupled with a plastic capillary hose serving as the outflow channel. All openings in the unit are sealed with oil leak-proof sealant. Following complete curing of the sealant, distilled water is injected into each channel to evaluate the integrity of the seal. The sealing operation should be repeated and verified until the device achieves complete tightness. Subsequently, the device should be affixed to the glass sheet using epoxy resin and allowed to stand for 30 min to ensure proper curing of the adhesive. The device is then stored in a cool and dry environment.

#### 2.1.2 Preparation of continuous phase and dispersed phase

##### 2.1.2.1 The continuous phase

In a beaker, 50 mL of vegetable oil was placed along with 20 μL of Tween 20, a surfactant. Stirring was performed to ensure complete mixing of the Tween 20 and vegetable oil. Subsequently, the beaker was positioned in a vacuum oven, evacuated to a vacuum, and allowed to stand for 30 min to remove air bubbles to obtain the continuous phase fluid required for the experiment.

##### 2.1.2.2 The dispersed phase

7 mL of deionized water and 350 mg of gelatin (Gum Power: 25 g, Bloom, Aladdin, China) were combined to generate an aqueous gelatin solution. 3 mL of pure water and 20 mg of photoinitiator (2-hydroxy-4′-(2-hydroxyethoxy)-2-methylpropiophenone, Shanghai, China, Aladdin) were mixed in a beaker to make a photoinitiator solution. The gelatin aqueous solution and photoinitiator solution were mixed and 0.1 mL of acid-base buffer was added to obtain the gelatin aqueous solution of photoinitiator. 0.9 mL of aqueous gelatin solution with photoinitiator and 0.1 mL of 25% Fe_3_O_4_ dispersion (20 nm) were placed in 8 test tubes. Hesperidin (Aladdin, Shanghai, China) powder was dissolved in dimethyl sulfoxide (DMSO, purity ≥99%, Sigma-Aldrich) and added to 8 test tubes to configure the final concentrations of 3.125, 6.25, 12.5, 25, 50, 100, 200, and 400 μM (the final DMSO concentration not exceeding 0.1%) of the dispersed phase fluid.

#### 2.1.3 Preparation of magnetic gelatin-hesperidin microrobots

The continuous phase and dispersed phase prepared above were individually injected into the microfluidic chip using syringe pumps. The flow rates of the two syringe pumps were adjusted, and both pumps were activated simultaneously (Wang et al., 2019). Dispersed phase and continuous phase were injected into the flow focusing device from the syringe pumps. Upon achieving uniform microdroplet formation at the device outlet, the uncured magnetic gelatin-hesperidin microrobots were collected in a beaker positioned at the outlet of the microchannel. The beaker was exposed to UV light for 20 min to facilitate the curing of the hydrogel. The cured gelatin-hesperidin microspheres were transferred to a test tube and subjected to centrifugation three times to eliminate the oil phase. Finally, the magnetic gelatin-hesperidin microrobots were shielded with tin foil and stored in a refrigerator at 4°C.

#### 2.1.4 Rotating magnetic field production

A three-dimensional Helmholtz coil, a multifunctional data collector, and three single-channel output power amplifiers were assembled to generate an external magnetic field. During the experiment, the cell culture dish was placed in the center of a three-dimensional Helmholtz coil, where the magnetic field intensity was evenly distributed. After adding the gelatin-hesperidin microrobots, the magnetic field was activated to propel the microrobot towards the cells.

### 2.2 Characterization of magnetic gelatin-hesperidin microrobots

#### 2.2.1 Observations on particle size

The cured gelatin-hesperidin microrobots were positioned on clean and dry slides, and the inverted microscope was adjusted to observe each microsphere individually. The maximum diameter of each gelatin-hesperidin microrobots was documented, and the particle size data obtained were subjected to statistical analysis. Subsequently, the fluorescent light source of the microsphere was activated, and the exposure time was adjusted to capture a clear fluorescent image in the dark field.

#### 2.2.2 Controlled motion

Gelatin-hesperidin microrobots with a diameter of about 30 μm were selected under a microscope and moved to the center of the field of view. By adjusting the strength and frequency of the exogenous rotating magnetic field, the microsphere was moved back and forth along a straight line in the field of view. After the microsphere movement was stabilized, photos and videos were taken and plotted.

#### 2.2.3 Drug release under near-infrared light irradiation

The microrobots of appropriate size were selected for the experiment. Photographs of clear microspheres in bright field and dark field were taken separately. Then every 30s, the photos under fluorescence were taken separately to obtain the images of microspheres releasing drugs by heat under near-infrared light irradiation.

#### 2.2.4 *In vitro* drug release study

High performance liquid chromatography (HPLC) was utilized to evaluate the degree of hesperidin release from gelatin-hesperidin microrobots. 1mL of gelatin-hesperidin microrobot was added to 5 mL of simulated body fluid (SBF, Gibco; Thermo Fisher Scientific, Inc.) and agitated in an incubator at 37°C and 40 rpm. The supernatant was extracted, centrifuged and filtered through a 0.22-μm membrane at 0, 5, 10, 15, 20, 25 and 30 min, then the sample was injected onto a Promosil 230 C18 column for analysis. The mobile phase was acetonitrile-water (20:80) and the detection wavelength was 285 nm.

### 2.3 Cell experiment

#### 2.3.1 Cell culture

To accurately replicate the high-glucose environment *in vitro*, a high-glucose DMEM medium (Gibco; Thermo Fisher Scientific, Inc) containing 15% fetal bovine serum and 1% penicillin-streptomycin, with a glucose concentration of 25 mmol/L, was prepared for cell culture and subsequent experiments. HFF-1 cells were provided by Mason Cell Centre (Zhejiang, China), cultured in high-glucose DMEM medium and maintained in a standard humidified incubator at 37°C with 5% CO_2_. The medium was refreshed as needed based on cell growth. Cell passaging was conducted when cell density reached 90%. Subsequent experiments were conducted after cells had undergone 3-4 generations of passaging.

#### 2.3.2 CCK8

The proliferation of HFF-1 cells at different time points was assessed using the CCK8 method. Initially, HFF-1 cells (5000 cells/well) were seeded in 96-well culture plates and cultured at 37°C, 5% CO_2_, and 90% humidity for 24 h. After incubation, cell morphology was examined under a light microscope. Subsequently, various concentrations of hesperidin solution (ranging from 3.125 μM to 400 μM) were added to the wells (100 μL each), with three replicate wells per concentration. Additionally, three replicate wells of a drug-free control group were included. The plates were then incubated at 37°C and 5% CO_2_ for 12 and 24 h respectively. Following incubation, 10 μL of CCK-8 solution (C0038, Beyotime) was added to each well and incubated for 4 h under light protection. Absorbance was measured at 450 nm using a microplate reader, and cell counts were determined after trypsin digestion. Cell viability and the number of treated cells were expressed as a percentage relative to the control. Data analysis was performed using Excel and GraphPad Prism. All experiments were performed in triplicate.

#### 2.3.3 Colony formation assay

The results of the CCK8 experiments indicated that HFF-1 cells exhibited optimal proliferation under the influence of hesperidin at a concentration of 25 μM. Therefore, the 25 μM concentration was selected for this experiment. HFF-1 cells (1 × 10^3^) were cultured in a 6-well culture plate for 24 h. Three groups were established, including a negative control (NC), hesperidin solution (25 μM), and magnetic gelatin-hesperidin microrobots (25 μM). Administration was conducted on days 2, 4, and 6, respectively, with each treatment lasting for 6 h. After 10 days, the cells were fixed with ethanol for 30–60 min and rinsed several times with PBS. Crystal violet staining solution (1 mL) was then added to each well for 10–20 min, followed by multiple PBS washes, air-drying, and photographic documentation using a digital camera.

#### 2.3.4 Cell scratch assay

First, horizontal lines were drawn behind the 6-well plate using a marker pen, ensuring an even spacing of approximately one line every 0.5–1 cm across the wells. Subsequently, about 5×10^5^ cells were added to each well, followed by incubation in a 37°C, 5% CO_2_ environment for 24 h. The next day, the tip of the pipettor was utilized to scratch the cells as perpendicular as possible to the horizontal lines drawn behind. Subsequently, the cells were washed three times with PBS to eliminate the scratched cells, and serum-free medium was replenished. Three groups were established, including a negative control (NC), hesperidin solution (25 μM), and magnetic gelatin-hesperidin microrobots (25 μM). Following drug administration, the cells were placed in a 37°C, 5% CO_2_ incubator for incubation. Samples were collected at 0, 6, and 12 h, and cell migration at specific locations was observed using an inverted microscope and documented. Upon opening the images using ImageJ software, the cell migration rate was computed.

### 2.4 Statistical analysis

All data were shown as means ± SD via at least triplicate samples. A two-tailed, Student’s t-test was used for testing the significance between two groups. Statistical analyses were performed using GraphPad PRISM 9 (GraphPad Software, Inc.) A one-way analysis of variance (ANOVA) with Dunnett’s test was performed to test the significance for multiple comparisons. A statistical significance was assumed at *p* < 0.05.

## 3 Results and discussion

### 3.1 Synthesis and characterization of magnetic gelatin-hesperidin microrobots

**FIGURE 1:**
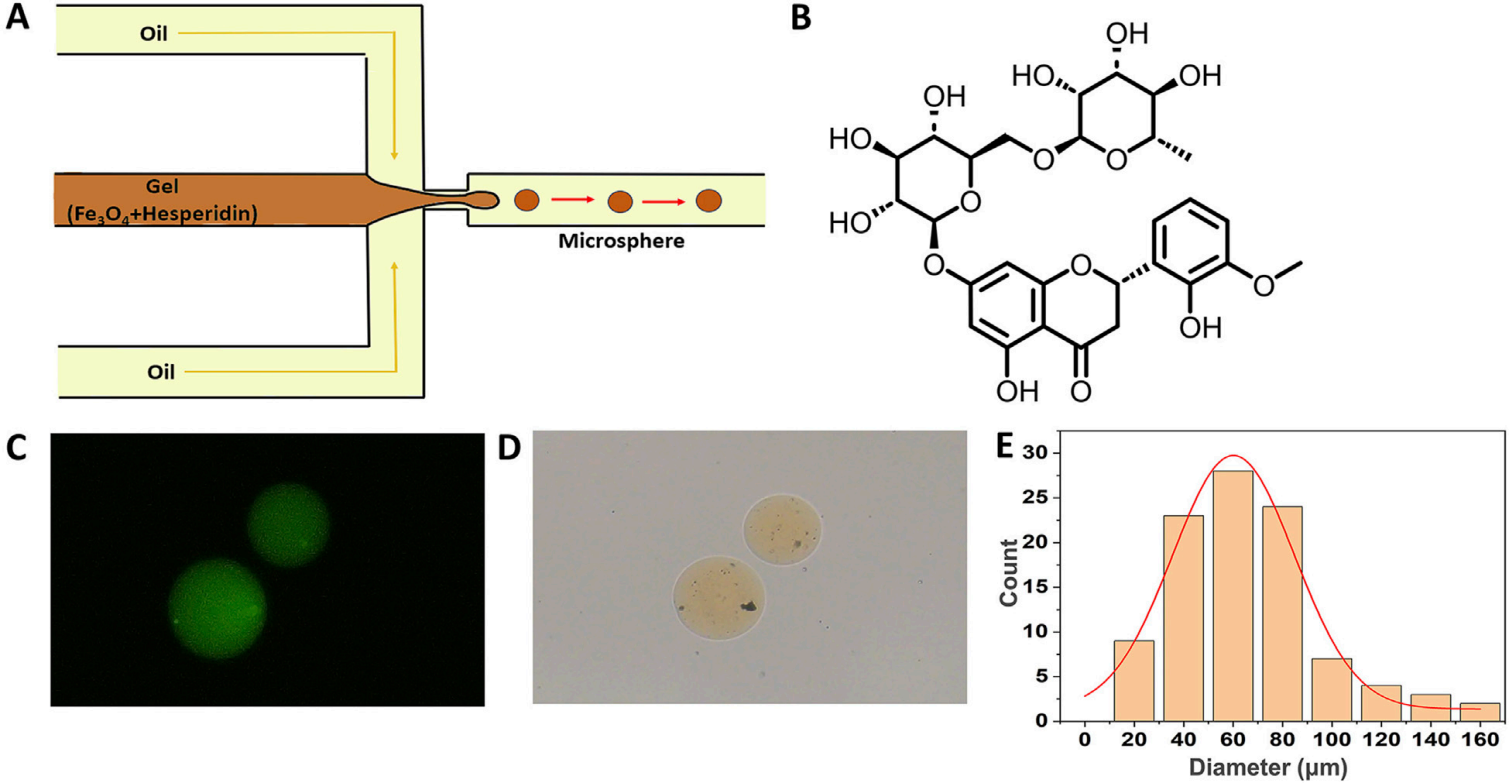
Fabrication and characterization of magnetically drivable gelatin-hesperidin microrobots. **(A)** The fabrication of magnetic gelatin-hesperidin microrobots using microfluidic chip. **(B)** The chemical formula structure of hesperidin. **(C)**The optical microscopy image of magnetic gelatin-hesperidin microrobots. scale bar 50 μm. **(D)** The fluorescence microscopic images of magnetic gelatin-hesperidin microrobots. scale bar 50 μm. **(E)** Particle size distribution of magnetic gelatin-hesperidin microrobots.

To produce the magnetic gelatin-hesperidin microrobots, we initially devised and manufactured a microfluidic chip. In this water-in-oil (W/O) system (Figure 1A), small droplets are generated through the combined effects of inertia and surface tension, facilitated by the shear force of continuous flow (Li et al., 2018). The continuous phase fluid is evenly distributed on both sides, while the dispersed phase fluid in the center is focused and sheared by the continuous phase fluid along symmetric paths, resulting in the generation of micro-droplets. Subsequently, UV irradiation is applied for curing, ultimately producing the desired hydrogel microrobots. In this experiment, various concentrations of hesperidin solution, Fe_3_O_4_ nanoparticles, and aqueous gelatin solution containing fluorescent particles were employed as the dispersed phase fluid, while vegetable oil with surfactant served as the continuous phase fluid. In a flow focusing device, the microdroplet generation rate and size can be adjusted by modifying the flow rate ratios of the continuous-phase and dispersed-phase fluids, as well as by varying their viscosities and the sizes of the channels (Park et al., 2016).

Figure 1B presents the chemical formula structure of hesperidin. Under the optical microscope, the prepared magnetic gelatin-hesperidin microrobots (Figure 1C) exhibit a yellow spherical geometry with no surface aggregation, appearing smooth and homogeneous. These observations indicate that the microrobots generated using this flow focusing device possess favorable morphology. The microrobots are observed to fluoresce green under a fluorescence microscope (Figure 1D) and this result indicates that hesperidin has been successfully loaded. The particle size distribution is determined by statistically analyzing the collected data and calculating the mean and standard deviation. Figure 1E demonstrates that the average diameter of the prepared microrobots is 76.15 μm, with a distribution spanning approximately 60–80 μm and a standard deviation of 29.14 μm. These results also imply a certain degree of monodispersity and normal distribution patterns for the microrobots. The particle size distribution and surface morphology of microspheres are critical parameters for their characterization and serve as primary factors influencing the drug release rate (Peng et al., 2012). Based on these characteristics, various dosage forms of drugs can be tailored, such as oral or topical formulations (Sitti and Wiersma, 2020).

**FIGURE 2:**
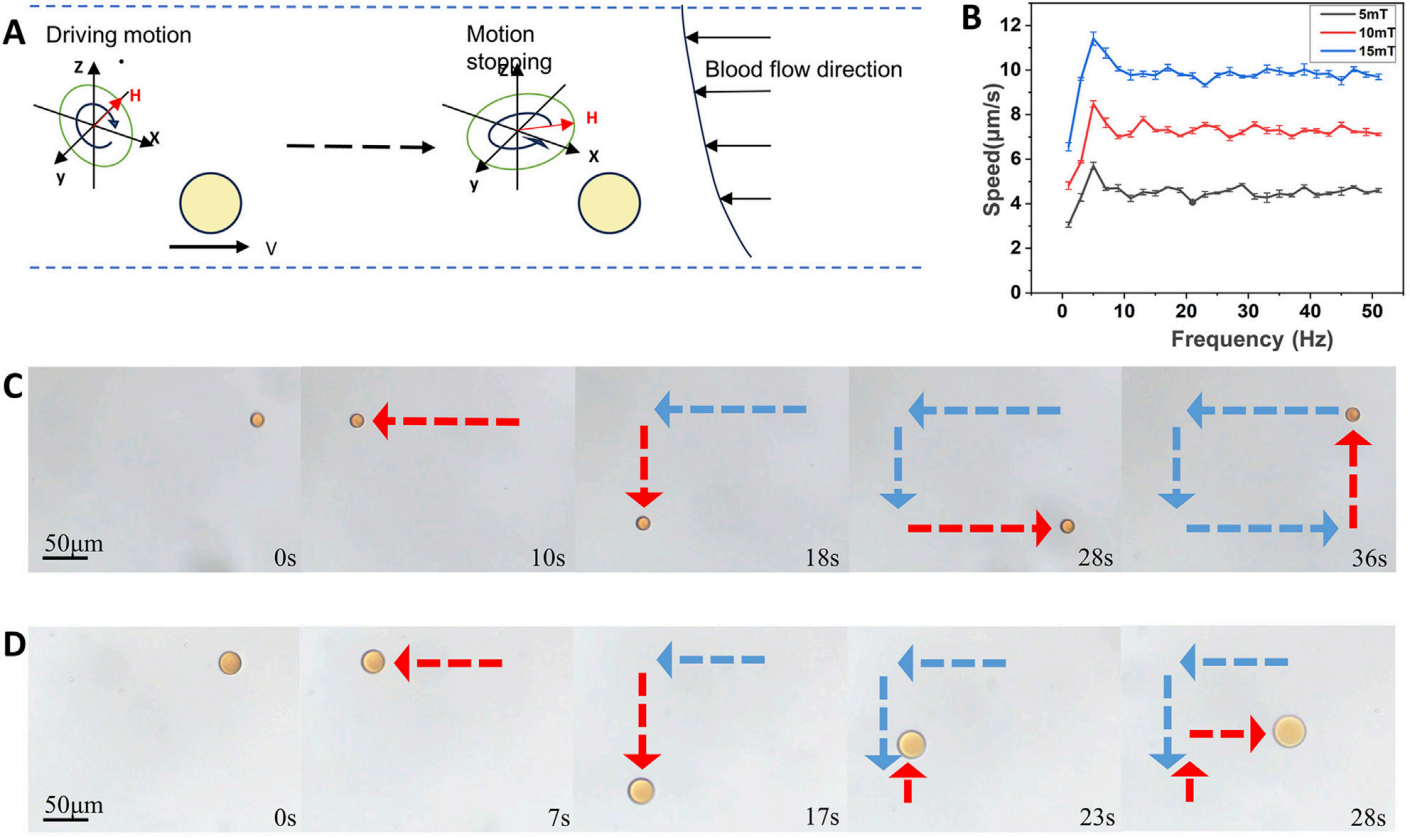
Directed motion of the hesperidin-gelatin microrobots under the control of a rotating magnetic field. **(A)** Change of the state of movement of the microrobots caused by changing the magnetic field. **(B)** The velocity of microrobots at different magnetic field strengths and frequencies. **(C)** Controllable motion of microrobots by rectangle. scale bar 50 μm. **(D)** Controllable motion of microrobots by letter F. scale bar 50 μm.

In this experiment, we devised and constructed a three-dimensional rotating magnetic field generation system using Helmholtz coils to produce a uniform magnetic field. The Helmholtz coil consists of a pair of identical coils positioned parallel and separated by R (where R represents the radius of the coil) (Saqib and AuthorAnonymous, 2020), through which the same rotational current passes (Ankamwar et al., 2010). This setup is capable of generating a uniform magnetic field near its axial center (Zhou et al., 2021). The magnetic field strength primarily depends on factors such as the number of turns, the size of the coil, and the intensity of the current passed through it. Fe_3_O_4_ nanoparticles are capable of moving in response to the magnetic field produced by an exogenous rotating magnetic field. Figure 2A illustrates the control strategy of three-dimensional rotating magnetic field generated by the three degrees of freedom Helmholtz coil and corresponding movement of microrobots.

For controlled motion performance experiments, we selected magnetic gelatin-hesperidin microrobots with a diameter of 50 μm. Figure 2B illustrates that under the influence of an exogenous magnetic field, the magnetic gelatin microrobots can achieve speeds of up to 11.411 μm/s by adjusting the strength and frequency of the magnetic field. Secondly, our observations indicate that the speed of the microrobots increases with the rise in magnetic field strength. Additionally, as the frequency increases, the speed of the microrobot under various magnetic field strengths initially rises to a certain threshold before declining and eventually stabilizing within a specific range. Controlling the motion speed of nanorobots enables the realization of more precise, effective, and safe drug delivery, treatment, and imaging. This advancement opens up possibilities for various applications in medical diagnosis and treatment, biosensing and imaging. We alter the motion direction of the microrobots by modifying the orientation of the exogenous rotating magnetic field. Motion trajectories of the microrobots are documented along a square path (Figure 2C and Supplementary Video S1) and a letter F shape (Figure 2D and Supplementary Video S2), driven by a magnetic field of 15 mT and 5 Hz. These advancements offer technical support for magnetically driven microrobots to navigate through complex fluids such as blood and traverse biological barriers.

**FIGURE 3:**
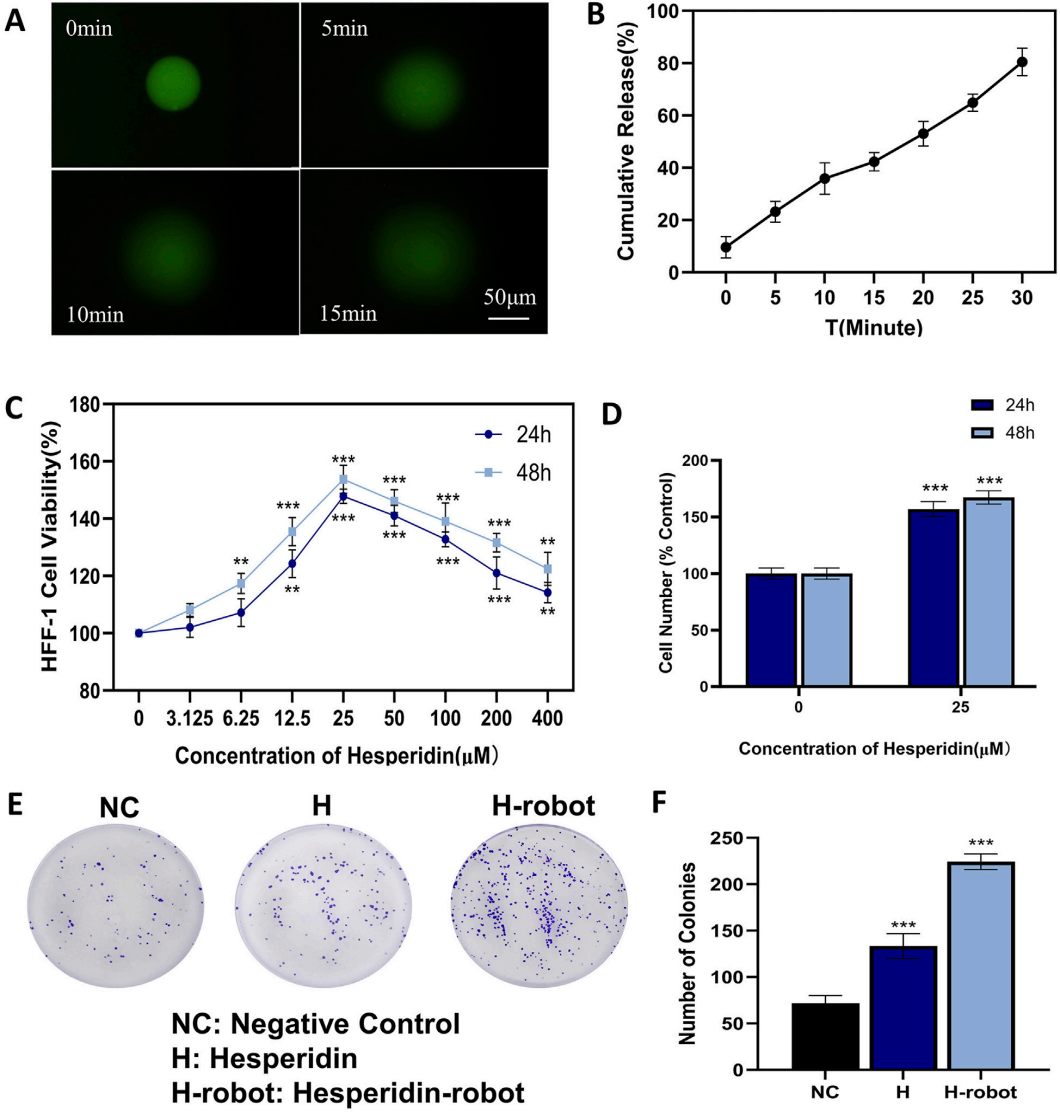
Effects of hesperidin and gelatin-hesperidin microrobots on the proliferation of HFF-1 cells under high sugar treatment. **(A)** Drug release profile of the hesperidin-gelatin microrobot under near-infrared light irradiation. Scale bar 50 μm. **(B)** Cumulative release profile of hesperidin from the gelatin-hesperidin microrobot over a period of 30 min **(C, D)** A CCK-8 assay was performed to measure HFF-1 cell viability following gelatin-hesperidin microrobots (0–400 µM) treatment. **(E, F)** The influence of hesperidin and gelatin-hesperidin microrobots on the colony formation of HFF-1 cells. *p < 0.05 and **p < 0.01 and ***p < 0.001 vs. NC group.

Hydrogel microrobots constructed with gelatin as the shell have been extensively investigated for controlled release applications (Kim et al., 2019). Upon reaching the target location, the hesperidin-gelatin microrobots experience a gradual increase in temperature over time when exposed to near-infrared light irradiation. Consequently, the gelatin shell undergoes gradual melting, leading to the eventual release of the encapsulated drug (Naidu and Paulson, 2011). Figure 3A shows the gelatin micro-nanobots under the fluorescence observation over time. The cumulative release profile of hesperidin is depicted in Figure 3B. We quantify the release rate of 1 mL of gelatin-hesperidin microrobots in 5 mL of SBF. Our findings reveal that the microrobot released 25.57% and 38.83% of hesperidin within the initial 5 and 10 min, while maintaining a stable release over a 30-min duration. Furthermore, no burst release is observed in this release profile. These findings suggest that the microrobots utilized in this experiment can achieve a slower and more sustained drug release rate, which is beneficial for maintaining plasma drug concentration and achieving the desired therapeutic effect (Aminu et al., 2020).

### 3.2 Impact of magnetic gelatin-hesperidin microrobots on the proliferation and migration capacity of fibroblasts

We conduct CCK8 and plate colony experiments to assess fibroblast growth under high glucose conditions (Omidfar et al., 2023). The findings (Figure 3C) from the CCK8 experiments reveal that the magnetic gelatin-hesperidin microrobots exhibit not only cytocompatibility but also a proliferative effect on cell growth. In the high glucose state, gelatin-hesperidin microrobots promote the growth of fibroblasts at both 12 and 24-h incubation times. Compared with the control group, the 25 μM gelatin-hesperidin microrobot groups exhibit nearly a 50% increase in the viability and number of HFF-1 cells (Figure 3D). Furthermore, the optimal concentration of 25 μM is selected for promoting fibroblast proliferation in plate colony experiments. As depicted in Figure 3E, the gelatin-hesperidin microrobot experimental group demonstrate a stronger promotion of fibroblast proliferative ability in the high glucose state compared to the experimental group treated with hesperidin alone. Additionally, it has been demonstrated that low concentrations of Fe3O4, as a component of magnetic microrobots, do not cause cell damage (Wu et al., 2020). The concentration of Fe3O4 in this experiment is 25 μg/mL across all experimental groups, suggesting no additional effects on the cells (Figure 3F).

**FIGURE 4:**
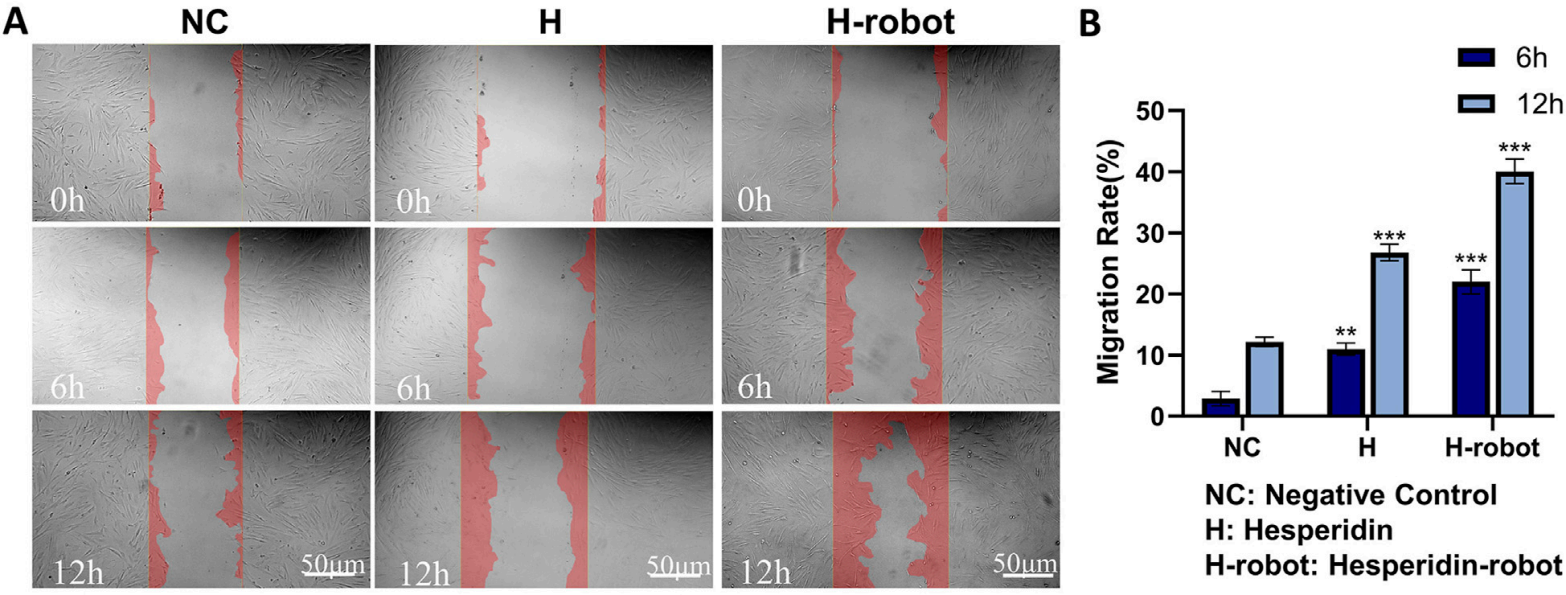
Effect of hesperidin and gelatin-hesperidin microrobots on HFF-1 cell mobility. **(A, B)** Wound healing assay of hesperidin and gelatin-hesperidin microrobots on HFF-1 cells at non-toxic concentrations (25µM, 6h, 12 h). Scale bar 50 μm. Magnification, ×100. **p < 0.01 and ***p < 0.001 vs. NC group.

The migration of fibroblasts is crucial for wound healing (Lerman et al., 2003). Therefore, we further investigated the effect of microrobots on the migration ability of fibroblasts in the high-glucose state using scratch experiments (Thulabandu et al., 2018). There are significant differences in the degree of migration when cells are scratched under different conditions. Compared with the control group, where significant cell migration is absent, the treatment group (Figure 4A) shows varying degrees of reduction in the scratch gap. After 12 h of incubation, the scratch gap of the gelatin-hesperidin microrobot group is significantly smaller than that of the treatment group with hesperidin alone. This suggests that the gelatin-hesperidin microrobot can significantly enhance the migration of fibroblasts. The quantitative analysis results (Figure 4B) reveal that the average migration rate of the gelatin-hesperidin microrobot group is 43.07%, significantly higher than that of the other groups. This further indicates that the gelatin-hesperidin microrobot group can enhance the regenerative migration ability of fibroblasts under high sugar conditions (Loots et al., 1999).

## 4 Conclusion

In conclusion, this study successfully develops a novel magnetic gelatin-hesperidin microrobot using microfluidics. The microrobot has a particle size of approximately 76.15 μm and exhibits biocompatibility and non-immunogenicity. Our data clearly indicates that it can serve as an effective micro-nanocarrier for the sustained delivery of hesperidin. Through adjustment of the peripheral magnetic field, the motion of the microrobots can be targeted and precisely controlled, facilitating the slow release of encapsulated drugs to enhance drug efficacy. Furthermore, cellular experiments have demonstrated its effectiveness in promoting the regeneration of fibroblasts under hyperglycemic conditions, as well as in restoring their migration ability impaired by hyperglycemia, thereby accelerating the healing of infected wounds. Furthermore, compared to direct drug administration, the gelatin-hesperidin micro-nano robots exhibit superior cellular promotion effects. Hence, this technology enables precise drug delivery and efficient utilization, suggesting that magnetic gelatin-hesperidin microrobots could serve as an effective novel therapeutic strategy for healing diabetic foot wounds.

## Data Availability

The raw data supporting the conclusions of this article will be made available by the authors, without undue reservation.
